# Molecular detection and whole genome characterization of Canine Parainfluenza type 5 in Thailand

**DOI:** 10.1038/s41598-021-83323-9

**Published:** 2021-02-16

**Authors:** Kamonpan Charoenkul, Chanakarn Nasamran, Taveesak Janetanakit, Supassama Chaiyawong, Napawan Bunpapong, Supanat Boonyapisitsopa, Ratanaporn Tangwangvivat, Alongkorn Amonsin

**Affiliations:** 1 Center of Excellence for Emerging and Re-Emerging Infectious Diseases in Animals, Faculty of Veterinary Science, Chulalongkorn University, Bangkok, Thailand; 2 Department of Veterinary Public Health, Faculty of Veterinary Science, Chulalongkorn University, Bangkok, 10330 Thailand; 3 Veterinary Diagnostic Laboratory, Faculty of Veterinary Science, Chulalongkorn University, Bangkok, Thailand

**Keywords:** Virology, Pathogens

## Abstract

Parainfluenza virus type 5 (PIV-5) causes respiratory infection in several animal species and humans. Canine parainfluenza virus type 5 (CPIV-5) causes respiratory disease in domestic dogs worldwide. In this study, we conducted a cross-sectional survey of CPIV-5 in dogs with respiratory symptoms from small animal hospitals in Thailand from November 2015 to December 2018. Our results showed that 32 out of 571 nasal swab samples (5.6%) were positive for CPIV-5 by RT-PCR specific to the NP gene. To characterize the viruses, three representative CPIV-5 were subjected to whole genome sequencing, and an additional ten CPIV-5 were subjected to HN, F, SH and V/P gene sequencing. Pairwise sequence comparison and phylogenetic analysis showed that Thai CPIV-5 was closely related to the CPIV-5 isolated from China and Korea. In conclusion, this study constitutes a whole genome characterization of CPIV-5 from dogs in Thailand. The surveillance of CPIV-5 should be further investigated at a larger scale to determine the dynamics, distribution and potential zoonotic transmission of CPIV-5.

## Introduction

Parainfluenza virus (PIV) is an enveloped, nonsegmented, single-stranded RNA virus. PIV-5 belongs to the family *Paramyxoviridiae*, genus *Rubulavirus*. The virus consists of seven genes encoding 8 proteins (F, HN, SH, M, NP, V, P, and L)^1^. PIV can be classified into 5 types, designated PIV 1–5. PIV-1 to PIV-4 can cause upper and lower respiratory tract infections in humans, especially in infants and young children^2–5^. PIV-5 has been reported to infect and cause respiratory disease in several host species.

PIV-5 was first isolated in 1956 from rhesus and cynomologus monkey kidney-cells^6^. The virus was previously named simian virus type 5 (SV-5) according to the host of isolation. Then, SV-5 was renamed to PIV-5 and prefixed according to the isolated species^7^. To date, the disease caused by PIV-5 in humans are still unclear. Some studies revealed that a virus serologically related to PIV-5 was associated with multiple sclerosis (MS), sclerosing panencephalitis (SSPE), Creutzfeldt-Jakob disease (CJD), pemphigus, atherosclerosis, Paget’s disease, hepatitis and common cold in humans^8–10^. There were in vitro studies and need to be identified as such PIV-5 was found in human respiratory cells and might impact human respiratory diseases^11,12^.

PIV-5 has been reported in several host species including pigs, cattle, dogs, hamsters, ferrets, monkeys, calves, lesser pandas and guinea pigs^10,13,14^. In pigs, PIV-5 co-infects with porcine reproductive and respiratory syndrome (PRRSV) and causes respiratory symptoms. In cattle, PIV-5 possibly causes severe respiratory illness and leads to a high morbidity rate in calves^15^. In dogs, canine parainfluenza virus type 5 (CPIV-5) was first isolated from dogs with respiratory signs in 1967 and was first named canine parainfluenza virus type 2 (CPIV-2) due to it causing a respiratory disease similar to that of human parainfluenza type 2 (HPIV-2)^16^. A subsequent study based on antigenic and sequence analyses revealed that CPIV-5 and HPIV-2 are different^17^. It has been reported that CPIV-5 is one of the common pathogens of canine infectious respiratory disease (CIRD). CPIV-5 causes mild to moderate respiratory illness in dogs. Dogs can develop severe clinical signs if co-infected with other respiratory viruses or bacteria^18–20^. In some cases, CPIV-5 can cause neurological disorders especially in puppies including encephalitis, seizures, myoclonus and posterior paresis^21,22^. The cross-species transmission of CPIV-5 has been reported in coyotes, ferrets and rodents^23,24^.

Interspecies transmission of PIV-5 between canines and humans has not been reported. However, a study suggested that PIV-5 might be a potential zoonotic pathogen^25^. Some studies have supported the hypothesis that genetic characteristics between PIV-5 isolated from canines and humans are highly similar with fewer nucleotide sequence variations (only 0.1% to 3% nucleotide difference)^7,26,27^. In addition, CPIV-5 can be grown in various cell lines especially human cell lines (2fTGH and HEp2) which might correlate with the host preference of the virus^28^. Since epidemiological and whole genome sequence information on CPIV-5 is still limited, in this study, we conducted a cross-sectional survey of CPIV-5 in dogs and characterized the whole genome of Thai CPIV-5.

## Results

In this study, we investigated canine parainfluenza type 5 (CPIV-5) infection in dogs with respiratory symptoms from November 2015 to December 2018. Our results showed that 5.6% (32/571) of nasal swab samples were positive for CPIV-5. From 3 years of surveillance, the highest occurrence of CPIV-5 was observed in November 2016 (41.7%), followed by December 2016 (33.3%) with statistical significance p < 0.05 when compared to other years (Supplement Table S1). Regarding the relationship between CPIV-5 infection and age group, the occurrences of CPIV-5 was statistically more frequent in dogs < 1 year (10.0%; 24/240) than in dogs older than 5 years (3.3%; 4/120) and dogs 1–4 years (1.9%; 4/211) (p = 0.0349 and 0.0003, p < 0.05), respectively. Regarding the relationship between CPIV-5 infection and vaccination history, the occurrence of CPIV-5 infection in dogs with incomplete CPIV-5 vaccination (10.4%; 28/269) was higher than in dogs fully vaccinated (1.3%; 4/302), with statistical significance (p < 0.05).

### Genetic characteristics of Thai canine parainfluenza type 5

**Figure 1:**
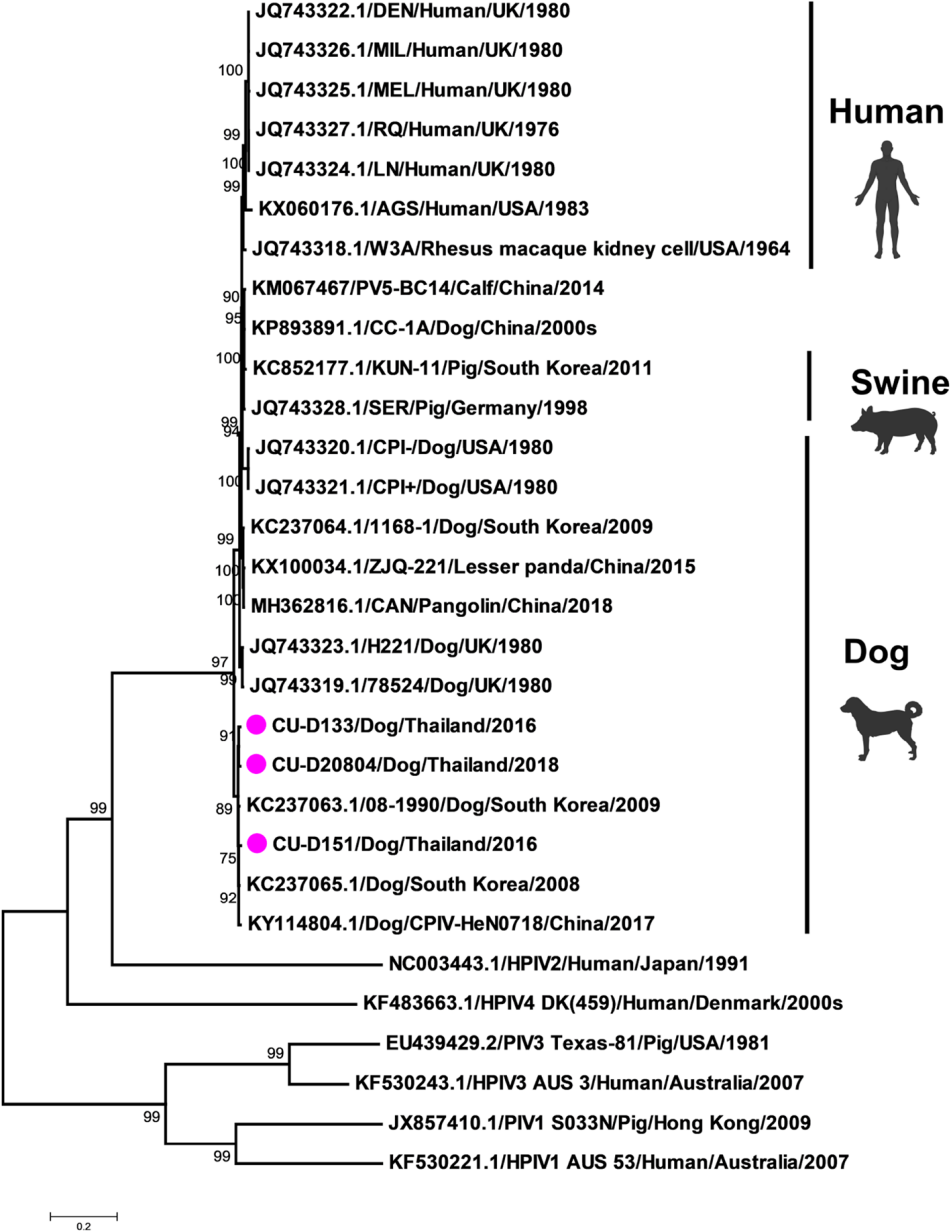
Phylogenic tree of the whole genome of Thai CPIV-5 and reference PIV1-5. Pink circles indicate Thai CPIV-5 in this study. The phylogenetic tree was constructed by using MEGA v.7.0 (Tempe, AZ, USA) with the neighbor-joining method with the Kimura 2-parameter with 1000 bootstrap replication^52^. The drawing was created by professional science figure service (BioRender.com).

In this study, we selected and characterized representatives of Thai CPIV-5 for whole genome sequencing (n = 3; CU-D133, CU-D151 and CU-D20804) and F, HN, V/P and SH gene sequencing (n = 10) (Table 1). Our results showed that the genome size of Thai CPIV-5 is 15,207 bp, containing seven genes as 3′-N-V/P-M-F-SH-HN-L-5′. Whole genome sequence analysis showed that Thai CPIV-5 possessed high nucleotide identity to the reference PIV5 with 96.1–99.4% nucleotide identities but low percentages of nucleotide identities with PIV-1 to PIV-4 (44.5–63.1% nucleotide identities). Comparing PIV-5, the whole genome of Thai CPIV-5 was closely related to Chinese CPIV-5 (HeN0718, 99.2% nucleotide identities) and Korean CPIV-5 (D277 and 08-1990, 99.4% and 99.2% nucleotide identities) (Table 2). For phylogenetic analysis, Thai CPIV-5 (n = 3) was grouped with PIV-5 from humans, pigs, dogs, lesser panda, and pangolins but separated from clusters of PIV-1 to PIV-4. The phylogenetic tree of the whole genome of PIV-5 could be divided into subgroups, e.g., human and simian subgroup, cattle and swine subgroup and canine subgroup. Thai CPIV-5 was grouped in the canine subgroup with CPIV-5 from China (HeN0718) and Korea (D277 and 08-1990) (Fig. 1).

**Table 1:** Description of canine parainfluenza type 5 (CPIV-5) characterized in this study.

Virus	Collection date	Age	Breed	Vaccination history	CPIV-5 detection	Sequencing	# GenBank
CU-D58	Jan 16	3 mts	Siberian Husky	I	+	F, HN, SH, V/P^a^	MT604002-05
CU-D103	Feb 16	2 mts	Bully	I	+	F, HN, SH, V/P	MT604006-09
CU-D133	Apr 16	> 7 years	Golden retriever	C	+	WGS^b^	MT603999
CU-D151	May 16	3 mts	Pomeranian	I	+	WGS	MT604000
CU-D373	Nov 16	3 mts	Pomeranian	I	+	F, HN, SH, V/P	MT604011-13
CU-D376	Dec 16	> 1 year	Mixed	I	+	F, HN, SH, V/P	MT604014-17
CU-D381	Dec 16	3 mts	Pekingese	I	+	F, HN, SH, V/P	MT604018-21
CU-D399	Jan 17	4 mts	Pomeranian	I	+	F, HN, SH, V/P	MT604022-25
CU-D400	Jan 17	7 mts	Pomeranian	I	+	F, HN, SH, V/P	MT604026-29
CU-D406	Jan 17	3 mts	Pomeranian	I	+	F, HN, SH, V/P	MT604030-33
CU-D466	Mar 17	2 mts	Mixed	I	+	F, HN, SH, V/P	MT604034-37
CU-D585	Sep 17	3 mts	Mixed	I	+	F, HN, SH, V/P	MT604038-41
CU-D20804	Feb 18	4 mts	Mixed	I	+	WGS	MT604001
CU-D361	Oct 16	2 mts	Pomeranian	I	+	−	−
CU-D369	Nov 16	6 mts	Pomeranian	I	+	−	−
CU-D370	Nov 16	10 years	Shih-Tzu	C	+	−	−
CU-D371	Nov 16	12 years	Poodle	C	+	−	−
CU-D372	Nov 16	4 mts	Pomeranian	I	+	−	−
CU-D377	Dec 16	> 1 year	Mixed	I	+	−	−
CU-D380	Dec 16	10 mts	Mixed	I	+	−	−
CU-D390	Dec 16	> 5 years	Mixed	C	+	−	−
CU-D483	Mar 17	> 1 year	Mixed	I	+	−	−
CU-D489	Mar 17	> 1 year	Mixed	I	+	−	−
CU-D493	Mar 17	6 mts	Mixed	I	+	−	−
CU-D497	Mar 17	6 mts	Mixed	I	+	−	−
CU-D20273	Dec 17	2 mts	Pomeranian	I	+	−	−
CU-D20277	Dec 17	2 mts	Bully	I	+	−	−
CU-D20364	Dec 17	3 mts	Pomeranian	I	+	−	−
CU-D20384	Jan 18	3 mts	Mixed	I	+	−	−
CU-D20803	Feb 18	3 mts	Mixed	I	+	−	−
CU-D21496	May 18	2 mts	Mixed	I	+	−	−
CU-D22309	Sep 18	3 mts	Samoyed	I	+	−	−

*C* complete vaccination, *I* incomplete vaccination.

^a^F, HN, SH, V/P; F, HN, SH, V/P gene sequencing.

^b^WGS; whole genome sequencing.

**Table 2:** Pairwise comparison of whole genome nucleotide sequences of Thai CPIV-5 (CU-D151) with reference parainfluenza viruses.

Virus	Accession no.	Host	Location	(%) Nucleotide identity								
				WGS	N (1530 nt)	F (1590–1656 nt)	HN (1698 nt)	SH (135 nt)	V (669 nt)	P (1177 nt)	M (1134 nt)	L (6768 nt)
CU-D151	This study	Canine	Thailand	100.0	100.0	100.0	100.0	(−)	100.0	100.0	100.0	100.0
CU-D133	This study	Canine	Thailand	99.1	99.3	99.0	99.5	(−)	99.0	98.9	99.4	99.3
CU-D20804	This study	Canine	Thailand	99.2	99.5	99.4	98.8	(−)	99.3	99.2	99.1	99.2
CU-D58	This study	Canine	Thailand	(−)	99.5	99.5	99.2	(−)	99.3	99.2	(−)	(−)
CU-D103	This study	Canine	Thailand	(−)	99.3	99.0	99.6	(−)	98.8	98.8	(−)	(−)
CU-D373	This study	Canine	Thailand	(−)	(−)	99.2	97.2	(−)	99.0	99.1	(−)	(−)
CU-D376	This study	Canine	Thailand	(−)	(−)	97.3	99.9	(−)	99.3	99.2	(−)	(−)
CU-D381	This study	Canine	Thailand	(−)	(−)	99.5	99.8	(−)	99.3	99.2	(−)	(−)
CU-D399	This study	Canine	Thailand	(−)	(−)	96.8	98.8	(−)	97.2	97.0	(−)	(−)
CU-D400	This study	Canine	Thailand	(−)	(−)	99.1	99.2	(−)	99.0	99.1	(−)	(−)
CU-D406	This study	Canine	Thailand	(−)	(−)	99.5	99.4	(−)	99.3	99.2	(−)	(−)
CU-D466	This study	Canine	Thailand	(−)	(−)	99.3	99.5	(−)	99.1	99.2	(−)	(−)
CU-D585	This study	Canine	Thailand	(−)	(−)	99.3	100.0	(−)	99.1	99.2		(−)
**Reference PIV-5**												
AGS	KX060176	AGS cell	USA	96.1	96.2	95.0	95.8	(−)	95.7	95.9	95.6	96.7
DEN	JQ743322	Human	UK	96.6	96.6	95.6	96.4	(−)	96.1	96.1	96.0	97.0
MIL	JQ743326	Human	UK	96.5	96.5	95.6	96.3	(−)	96.0	96.0	96.0	97.0
MEL	JQ743325	Human	UK	96.5	96.3	92.5	96.4	(−)	96.1	96.1	95.9	97.0
RQ	JQ743327	Human	UK	96.5	96.5	95.5	96.3	(−)	96.0	96.0	95.9	97.0
LN	JQ743324	Human	UK	96.5	97.2	95.5	96.3	(−)	96.0	96.0	95.9	97.0
W3A	JQ743318	Macaque cell	USA	97.0	97.3	92.0	96.9	(−)	96.9	96.7	95.9	97.6
HeN0718	KY114804	Canine	China	99.2	99.5	99.3	96.9	(−)	98.8	99.1	99.0	99.3
CC-14	KP893891	Canine	China	97.2	97.5	96.4	97.6	(−)	96.7	96.9	96.3	97.8
H221	JQ743323	Canine	UK	97.5	97.5	96.7	97.9	(−)	97.6	97.2	96.9	98.1
78524	JQ743319	Canine	UK	97.5	97.4	96.7	97.9	(−)	97.3	97.1	96.9	97.9
CPI +	JQ743321	Canine	USA	96.7	96.4	95.6	96.9	(−)	96.3	96.3	95.9	97.3
CPI-	JQ743320	Canine	USA	96.7	96.4	95.5	96.9	(−)	96.0	96.2	95.9	97.3
08-1990	KC237063	Canine	Korea	99.2	99.5	99.5	99.5	(−)	99.4	99.3	99.5	99.4
D277	KC237065	Canine	Korea	99.4	99.9	99.5	99.6	(−)	99.6	99.6	99.6	99.6
1168-1	KC237064	Canine	Korea	97.4	97.1	96.6	97.9	(−)	97.0	96.9	96.9	98.0
SER	JQ743328	Swine	Germany	97.2	97.3	96.4	97.6	(−)	96.7	96.9	96.4	97.7
KNU-11	KC852177	Swine	Korea	97.0	96.9	96.3	97.3	(−)	96.1	96.3	96.0	97.6
PV5-BC14	KM067467	Calve	China	97.2	97.1	96.4	97.5	(−)	96.6	96.7	96.4	97.7
ZJQ-221	KX100034	Lesser panda	China	97.3	96.9	96.5	97.6	(−)	97.0	96.9	96.7	97.9
**Other reference PIV I to IV**												
HPIV-1	KF530221	Human	Australia	45.9	(−)	(−)	(−)	(−)	(−)	(−)	(−)	(−)
S033N	JX857410	Swine	Hong Kong	44.5	(−)	(−)	(−)	(−)	(−)	(−)	(−)	(−)
HPIV-2	NC003443	Human	Japan	63.1	(−)	(−)	(−)	(−)	(−)	(−)	(−)	(−)
HPIV-3	NC001796	Human	Australia	46.8	(−)	(−)	(−)	(−)	(−)	(−)	(−)	(−)
Texas-81	EU439429	Swine	USA	45.8	(−)	(−)	(−)	(−)	(−)	(−)	(−)	(−)
HPIV-4	KF483663	Human	Denmark	52.1	(−)	(−)	(−)	(−)	(−)	(−)	(−)	(−)

**Figure 2:**
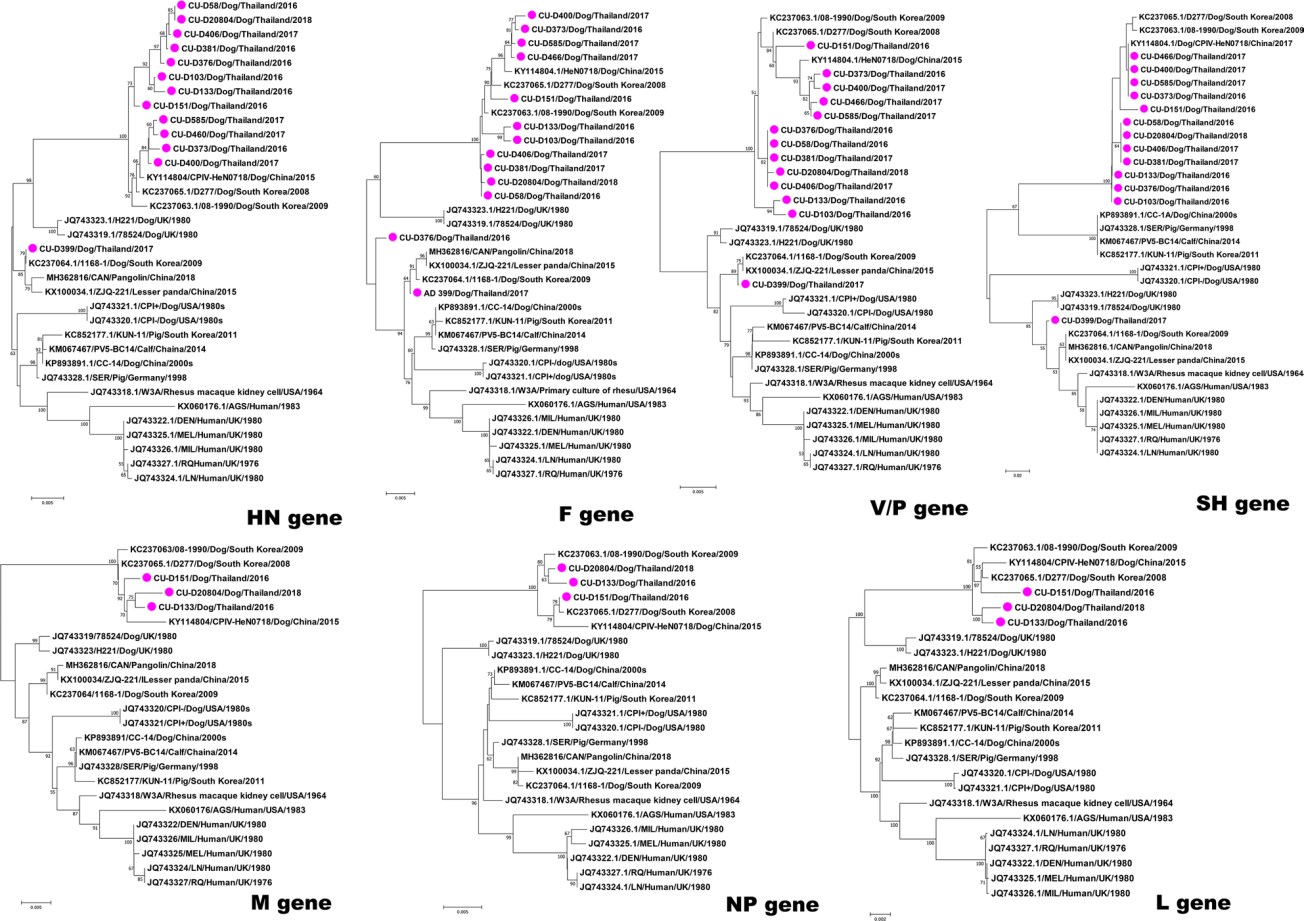
Phylogenetic trees of the HN, F, M, V/P, NP, and L genes of Thai CPIV-5 and reference PIV-1-5. Pink circles indicate Thai CPIV-5 in this study. The phylogenetic tree was constructed by using MEGA v.7.0 (Tempe, AZ, USA) with the neighbor-joining method with the Kimura 2-parameter with 1000 bootstrap replication^52^.

Pairwise comparison of nucleotide sequences showed that the HN, F, V/P and SH genes of Thai CPIV-5 possessed high nucleotide identities to Chinese CPIV-5 (HeN0718; 96.9–99.5%) and Korean CPIV-5 (D277 and 08-1990; 99.3–99.9%), which were similar to the whole genome sequences (Table 2). The phylogenetic analysis of the F, HN, and V/P genes showed that Thai CPIV-5 was grouped with Chinese CPIV-5 (HeN0718) and Korea CPIV-5 (D277 and 08-1990) (Fig. 2). Moreover, the M, NP and L genes of Thai CPIV-5 (CU-D131, CU-D151 and CU-D20804) had the highest nucleotide identities to Korean CPIV-5 (D277; 99.6–99.9%). The phylogenetic analysis results showed that the M, NP and L genes were also closely related to CPIV-5 from Chinese and Korean strains (Fig. 2).

Genetic analysis of the HN gene (1698 nucleotides, 565 amino acids) of Thai CPIV-5 showed that amino acid residues at the receptor binding site (positions 186–190) and cleavage site (positions 390 and 523) of the HN protein contained QDHVS (186–190), E390 and Y523. Amino acid residues at the stalk regions contained S60, Y77, L90, E91 and Q102 identical to the reference PIV-5. Amino acid residues at positions 37, 342, 437, and 457, which correlated with neutralizing antibodies, contained E37, K342, T437, and F457. It is noted that Q342K was only observed in all Thai CPIV-5, which was identical to CPIV-5 from China (HeN0718) and Korea (D277 and 08-1990) but not in other CPIV-5 and human PIV-5 (Table 3). Amino acid residues related to host preference (human specific) at I22L, A49S, R57G, T254A, N318S, K460T and M536T were analyzed. Thai CPIV-5 contained I22, A49, R57, T254, N318, K460 and M536, which are not human specific amino acids. Unique amino acids for Thai, Chinese and Korean CPIV-5 were also observed at T19I, K43E, T62I, T141A, F252L, F353L and G446R suggesting unique subclustered characteristics (Table 4).

**Table 3:** Genetic analysis of the HN gene of Thai CPIV-5 and reference PIV-5 at the receptor binding, cleavage site and stalk region.

Virus	Host	HN gene											
		HN gene				Receptor binding site	Cleavage site		HN stalk				
		37	342	437	457	186–190	390	523	60	77	90	91	102
**Reference PIV-5**													
AGS	AGS cell	E	K	T	A	QDHVS	E	Y	S	Y	L	E	H
W3A	Macaque cell	E	Q	T	F	QDHVS	E	Y	S	Y	L	E	Q
DEN	Human	E	Q	T	F	QDHVS	E	Y	S	Y	L	E	Q
MIL	Human	E	Q	T	F	QDHVS	E	Y	S	Y	L	E	Q
MEL	Human	E	Q	T	F	QDHVS	E	Y	S	Y	L	E	Q
RQ	Human	E	Q	T	F	QDHVS	E	Y	S	Y	L	E	Q
LN	Human	E	Q	T	F	QDHVS	E	Y	S	Y	L	E	Q
SER	Swine	E	Q	T	F	QDHVS	E	Y	S	Y	L	E	Q
KNU-11	Swine	E	Q	T	F	QDHVS	E	Y	S	Y	L	E	Q
PV5-BC14	Calve	E	Q	T	F	QDHVS	E	Y	S	Y	L	E	Q
ZJQ-221	Lesser panda	E	Q	T	F	QDHVS	E	Y	S	Y	L	E	Q
CAN	Pangolin	E	Q	T	F	QDHVS	E	Y	S	Y	L	E	Q
H221	Canine	E	Q	T	F	QDHVS	E	Y	S	Y	L	E	Q
78524	Canine	E	Q	T	F	QDHVS	E	Y	S	Y	L	E	Q
CPI +	Canine	E	Q	T	F	QDHVS	E	Y	S	Y	L	E	Q
CPI-	Canine	E	Q	T	F	QDHVS	E	Y	S	Y	L	E	Q
08-1990	Canine	E	K	T	F	QDHVS	E	Y	S	Y	L	E	Q
D277	Canine	E	K	T	F	QDHVS	E	Y	S	Y	L	E	Q
1168-1	Canine	E	Q	T	F	QDHVS	E	Y	S	Y	L	E	Q
CC-14	Canine	E	Q	T	F	QDHVS	E	Y	S	Y	L	E	Q
HeN0718	Canine	E	K	T	F	QDHVS	E	Y	S	Y	L	E	Q
**This study**													
CU-D58	Canine	E	K	I	F	QDHVS	E	Y	S	Y	L	E	Q
CU-D103	Canine	E	K	T	F	QDHVS	E	Y	S	Y	L	E	Q
CU-D133	Canine	E	K	T	F	QDHVS	E	Y	S	Y	L	E	Q
CU-D151	Canine	E	K	T	F	QDHVS	E	Y	S	Y	L	E	Q
CU-D373	Canine	E	K	T	F	QDHVS	E	Y	S	Y	L	E	Q
CU-D376	Canine	E	K	I	F	QDHVS	E	Y	S	Y	L	E	Q
CU-D381	Canine	E	K	T	F	QDHVS	E	Y	S	Y	L	E	Q
CU-D399	Canine	E	Q	T	F	QDHVS	E	Y	S	Y	L	E	Q
CU-D400	Canine	E	K	T	F	QDHVS	E	Y	S	Y	L	E	Q
CU-D406	Canine	E	K	T	F	QDHVS	E	Y	S	Y	L	E	Q
CU-D466	Canine	E	K	T	F	QDHVS	E	Y	S	Y	L	E	Q
CU-D585	Canine	E	K	T	F	QDHVS	E	Y	S	Y	L	E	Q
CU-D20804	Canine	E	K	T	F	QDHVS	E	Y	S	Y	L	E	Q

**Table 4:** Genetic analysis of the HN gene of Thai CPIV-5 and reference PIV-5 at the human-specific residues.

Virus	Host	Location	Primate specific amino acid						Lineage specific amino acid^a^							
			22	49	57	254	318	460	536	19	43	62	141	252	353	446
**Reference PIV-5**																
AGS	AGS Cell		L	S	G	A	S	T	T	T	K	T	T	F	F	G
DEN	Human	UK	L	S	G	A	S	T	T	T	K	T	T	F	F	G
MIL	Human	UK	L	S	G	A	S	T	T	T	K	T	T	F	F	G
MEL	Human	UK	L	S	G	A	S	T	T	T	K	T	T	F	F	G
RQ	Human	UK	L	S	G	A	S	T	T	T	K	T	T	F	F	G
LN	Human	UK	L	S	G	A	S	T	T	T	K	T	T	F	F	G
W3A	Macaque cell		I	A	R	A	N	T	M	T	K	T	T	F	F	G
SER	Swine	Germany	I	A	R	T	N	K	M	T	K	T	T	F	F	G
KNU-11	Swine	South Korea	I	A	R	T	N	K	M	T	K	T	T	F	F	G
PV5-BC14	Calve	China	I	A	R	T	N	K	M	T	K	T	T	F	F	G
ZJQ-221	Lesser panda	China	I	A	R	T	N	K	M	T	K	T	T	F	F	G
CAN	Pangolin	China	I	A	R	T	N	K	M	T	K	T	T	F	F	G
H221	Canine	UK	I	A	R	T	N	K	I	T	K	T	T	F	F	G
78524	Canine	UK	I	A	R	T	N	K	M	T	K	T	T	F	F	G
CPI +	Canine	USA	I	A	R	T	N	K	M	T	K	T	T	L	F	G
CPI-	Canine	USA	I	A	R	T	N	K	M	T	K	T	T	L	F	G
08-1990	Canine	South Korea	I	A	R	T	N	K	M	I	E	I	A	L	L	R
D277	Canine	South Korea	I	A	R	T	N	K	M	I	E	I	A	L	L	R
1168-1	Canine	South Korea	I	A	R	T	N	K	M	T	K	T	T	F	F	G
CC-14	Canine	China	I	A	R	T	N	K	I	T	K	T	T	F	F	G
HeN0718	Canine	China	I	A	R	T	N	K	M	I	E	I	A	L	L	R
**This study**																
CU-D58	Canine	Thailand	I	A	R	T	N	K	M	I	E	I	A	L	L	R
CU-D103	Canine	Thailand	I	A	R	T	N	K	M	I	E	I	A	L	L	R
CU-D133	Canine	Thailand	I	A	R	T	N	K	I	I	E	I	A	L	L	R
CU-D151	Canine	Thailand	I	A	R	T	N	K	M	I	E	I	A	L	L	R
CU-D373	Canine	Thailand	I	A	R	T	N	K	M	I	E	I	A	L	L	R
CU-D376	Canine	Thailand	I	A	R	T	N	K	M	I	E	I	A	L	L	R
CU-D381	Canine	Thailand	I	A	R	T	N	K	M	I	E	I	A	L	L	R
CU-D399	Canine	Thailand	I	A	R	T	N	K	M	T	K	T	I	F	F	G
CU-D400	Canine	Thailand	I	A	R	T	N	K	M	I	E	I	A	L	L	R
CU-D406	Canine	Thailand	I	A	R	T	N	K	M	I	E	I	A	L	L	R
CU-D466	Canine	Thailand	I	A	R	T	N	K	M	I	E	I	A	L	L	R
CU-D585	Canine	Thailand	I	A	R	T	N	K	M	I	E	I	A	L	L	R
CU-D20804	Canine	Thailand	I	A	R	T	N	K	M	I	E	I	A	L	L	R

^a^Lineage: CPIV-5 sublineage; Thai, Chinese, and Korean sublineages.

Genetic analysis of the F gene showed a low level of genetic variation. Amino acid residues related to host preference (human specific) were observed at T3I, S19G, I301M, T438S, L498F, S530Q and R536Q. One Thai CPIV-5 (CU-D151) contained R536Q similar to some human PIV-5 (DEN, MIL, RQ, and LN). Moreover, Thai CPIV-5 contained 22P and 443P, which were similar to PIV-5 from humans and pigs suggesting potential human preference characteristics^29–31^ (Supplement Table S2). Genetic analysis of the V/P gene showed that amino acids related to viral RNA synthesis contained S157, T286 and K254 similar to most CPIV-5 (Supplement Table S3).

**Figure 3:**
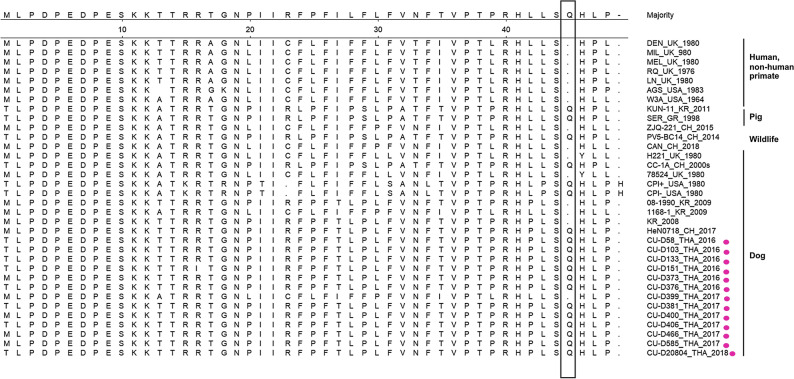
Alignment of deduced amino acids of the SH gene of Thai CPIV-5 and reference PIV-5 viruses. The box indicates amino acid substitution at the stop codon (Q). Pink circles indicate Thai CPIV-5 in this study.

Genetic analysis of the SH gene showed that Thai CPIV-5 (CU-D58, CU-D103, CU-D133, CU-D151, CU-D376, CU-D381, CU-D406, and CU-D20804) contained a non-synonymous substitution at the start codon (M1T). Distinct nucleotide substitutions at T133C were observed and resulted in the extension of four amino acids at the stop codon, similar to those of CPIV-5 from China and Korea. Thus, the SH protein of Thai, Chinese, and Korean CPIV-5 is four amino acids longer than that of the reference PIV-5 (Supplement Table S3 and Fig. 3).

## Discussion

Parainfluenza virus type 5 (PIV-5) can infect and cause respiratory diseases in various mammals. Canine parainfluenza virus type 5 (CPIV-5) is highly contagious and causes mild to moderate respiratory diseases in dogs worldwide. Coinfection with CPIV-5 and other viruses or bacteria can cause more virulent clinical signs. This study revealed the occurrence of CPIV-5, which was relatively high during the winter season in Thailand (November to January). A similar finding of high occurrence detected in the cold season has also been reported^32^. CPIV-5 could be detected in younger dogs (< 1 year) more than in older dogs. Dogs of all ages could be infected with CPIV-5, but younger dogs (< 1 year) are more susceptible. This observation is in agreement with a previous report that CPIV-5 could be observed more in younger dogs than in dogs in other age groups^33–35^. Regarding vaccination history, CPIV-5 infection was higher in dogs with incomplete vaccination (10.4%) than in dogs with complete vaccination (1.32%). The CPIV-5 vaccine used in Thailand was modified live CPIV-5 combined with other pathogens (e.g., canine distemper virus, canine parvovirus and canine coronavirus). Some studies have suggested that vaccinated dogs can show mild clinical signs and shed the virus after infection^36^. It is noted that, the CPIV-5 characterized in this study was obtained from nasal swabs of dogs with and without vaccination. A previous study revealed that whole genome sequences of CPIV-5 vaccine was identical with PIV-5 strain W3A, which different from Thai-CPIV-5^37^. Moreover, all three Thai-CPIV-5 contained unique amino acids of Asian CPIV-5 subcluster and distinguished from W3A and CPIV-5 from the US and UK. Thus, it more likely that the CPIV-5 in this study were isolated from naturally infected dogs in Thailand.

To date, only nine whole genome sequences of CPIV-5 are available in the GenBank database. This study provided additional information on the whole genome sequences of CPIV-5 from Thailand (n = 3). Based on phylogenetic analysis of the whole genome, Thai CPIV-5 belongs to parainfluenza type 5 and subcluster CPIV-5 (canine sublineage) and is separated from swine and human sublineage. Within the canine sublineage, Thai CPIV-5 was closely related to CPIV-5 from South Korea (08-1990 and D227) and China (CPIV-HeN0718). Thai CPIV-5 had the highest nucleotide identities (99.4%) to CPIV-5 from Korea. Phylogenetic analyses of the HN, F, V/P, M, NP and L genes showed similar results, in which Thai CPIV-5 was grouped together with CPIV-5 from Korea (08-1990 and D227) and China (CPIV-HeN0718). One Thai CPIV-5 (CU-D399) was closely related to PIV-5 from the pangolin (CAN) and lesser panda (ZJQ-221), which was similar to CPIV-5 (1168-1 from Korea). Our results suggested that Thai CPIV-5 potentially originated from the same ancestor as CPIV-5 from China and South Korea. Similarly, a unique cluster of CPIV-5 from dog in China (CC-1A, 2000s), PIV-5 from calf in China (PV5-BC14, 2014) and PIV-5 from pig in Germany (SER, 1998) and South Korea (KUN-11, 2011) was observed suggesting potential common ancestor of these viruses and required further investigations.

Thai CPIV-5 contained no amino acid mutations in the HN protein at the receptor binding site, cleavage site or HN stalk region. It has been reported that the amino acid residue at E37 is associated with virus entry into host cells by clathrin-coated pits and the endocytic pathway^38,39^. Amino acid residues at L90, E91, Q102, QDHVS (186-190), E390 and Y523 are associated with viral receptor binding of the viruses^40,41^. Amino acid residues at K342, T437, and F457 are associated with neutralizing antibodies^42^. In this study, some Thai CPIV-5 contained T437I (CU-D58 and CU-D376) and K342Q (CU-D399) which is similar to PIV-5 from dogs and humans. However, the importance of these mutations (T437I and K342Q) in neutralizing antibodies requires further investigation. A previous study reported that mutations in HN stalk regions might affect viral fusion to host cells^41,43^.

Thai CPIV-5 contained some host preference amino acid residues (human specific residues) in the F gene. For example, the amino acids at 22P and 443P in the F gene were observed in both Thai CPIV-5 and PIV-5 from humans and pigs^29–31^. One Thai CPIV-5 (CU-D151) also contained R536Q, similar to human PIV-5. For the V and P proteins, there was no amino acid mutation in Thai PCIV-5. It has been reported that amino acid mutations of S157F, K254 R and T286A of V and P proteins can result in high progeny virus production and the apoptosis of infected cells^44–46^. For the SH protein, Thai CPIV-5 contained an amino acid substitution at the start codon, which can also be observed in swine PIV-5, cattle PIV-5 and canine PIV-5. Mutation of the start codon can result in no expression of the SH protein^7,31^. The function of the SH protein is unclear, but some studies have reported an association with virus survival in host cells and control of host cell apoptosis^31,47,48^. It should be noted that Thai, Chinese and Korean CPIV-5 contained four amino acids longer than the reference PIV-5. Thus, the SH gene can be used as a genetic marker for the differentiation of Asian CPIV-5 from other CPIV-5.

In summary, this study is the first report of whole genome characterization of CPIV-5 in Thailand. Phylogenetic analyses showed that Thai CPIV-5 might have originated from a common ancestor with CPIV-5 from Korea and China. To date, there is no evidence of PIV-5 cross-species transmission between dogs and humans. However, it is imperative to educate pet owners, veterinarians and others who come into close contact with domestic dogs about zoonotic awareness. In Thailand, the surveillance of CPIV-5 should be further investigated on a larger scale to determine the dynamics, distribution and genetic characteristics of CPIV-5.

## Materials and methods

### Canine samples

From November 2015 to December 2018, a total of 571 nasal swab samples were collected from dogs with respiratory symptoms, including sneezing, nasal discharge, cough, and dyspnea. Sample collection was conducted at Chulalongkorn University’s Veterinary Teaching Hospital and private small animal hospitals in Bangkok, Thailand. The animal demographic data, including age, sex, breed, contact history, and vaccination history, were recorded. This study was conducted under approval from the Institute of Animal Use and Care Committee (IACUC# 1731074), and all procedures were completed in accordance with the relevant guidelines and regulations.

### Canine parainfluenza virus identification

RNA extraction from nasal swab samples was conducted by using the QIAmp viral RNA mini kit (Qiagen, Hilden, Germany) following the manufacturer’s recommendations. Briefly, 140 μl of nasal swab sample was lysed by Buffer AVL–carrier RNA and 560 μl of ethanol. The mixture was centrifuged and transferred into a column, and then 500 μl each of buffers AW1 and AW2 were added. Finally, the RNA was eluted by 50 μl of buffer AVE. RNA was stored at − 20 °C until use. CPIV-5 detection was performed by using a nested RT-PCR assay specific to the NP gene of PIV-5 (Supplement Table S4)^49^. Briefly, one-step nested RT-PCR was conducted in a total final volume of 25 μl comprised of 3 μl of template RNA, 12.5 μl of 2 × reaction mix, 0.6 μl of 10 μM forward (CPiV-F363) and reverse primer (CPiV-R538), 1.2 μl of SuperScript III RT (Invitrogen, USA) and distilled water to a final volume of 25 μl. The first round of PCR product was diluted 1:5 with distilled water and subjected to a second round by using the TopTaq Master Mix Kit (Qiagen, Germany). The final volume was 20 μl, including 10 μl of 2 × TopTaq Master Mix, 1 μl of 10 μM forward (CPiV-F428) and reverse primer (CPiV-R538), 2 μl of 10 × coral load, and 1 μl of DNA. For the first round of nested RT-PCR conditions, the reaction contained a cDNA synthesis step at 55 °C for 30 min, an initial denaturation step at 94 °C for 2 min, 40 cycles of denaturation at 94 °C for 30 s, annealing at 55 °C for 30 s and extension at 68 °C for 30 s, and a final extension step at 68 °C for 6 min. For the second round of nested PCR conditions, the reaction comprised an initial denaturation step at 94 °C for 3 min, 35 cycles of denaturation at 94 °C for 30 s, annealing at 55 °C for 30 s and extension at 72 °C for 30 s and a final extension step at 72 °C for 7 min. To confirm CPIV5, 4 μl of PCR product was run on a 1.5% agarose gel with red safe. The expected size of the positive CPIV-5 product was 188 bp. Statistical analysis by the Fisher’s exact test was used to compare the proportion of CPIV-5 positivity among dogs categorized by the time of sample collection, age of dogs, and vaccination history.

### Canine parainfluenza virus isolation

To isolate CPIV-5, RT-PCR-positive nasal swabs were subjected to virus isolation by using a Vero cell monolayer (ATCC, USA) at the Faculty of Veterinary Science, Chulalongkorn University. In brief, nasal swab sample were filtered with a 0.22 µm filter and inoculated onto a Vero cell monolayer containing Dulbecco’s minimal essential medium (DMEM, Gibco), 2% fetal bovine serum (FBS, Gibco), and gentamycin sulfate (50 μg/ml) at 37 °C in 5% CO_2_. If a cytopathic effect (CPE) was observed, the virus was harvested by centrifugation at 1000 rpm for 10 min. The cell suspension was then screened for CPIV-5 by using nested RT-PCR as previously described^49,50^. The isolated viruses were kept at − 80 °C for the pathogenesis studies in the future.

### Canine parainfluenza virus characterization

In this study, Thai-CPIV-5 was selected for either whole genome sequencing (n = 3) or F, HN, V/P, and SH gene sequencing (n = 10). The representative CPIV-5 was selected based on epidemiological and demographic data such as the age of the dog, date of isolation, breed, and vaccination history. For whole genome sequencing, nucleotide sequences of each virus gene were amplified by PCR using oligonucleotide primers specific to each gene. The primers were synthesized per previous report and newly designed by using Primer 3 plus (Supplement Table S4)^50,51^. Nucleotide sequencing was conducted at the 1^st^ Base Laboratories Sdn Bhd, Malaysia. The nucleotide sequences were validated and assembled by SeqMan software v.5 v.5.03 (DNASTAR Inc., Wisconsin, USA). In this study, nucleotide sequences of Thai CPIV-5 were submitted to the GenBank database under the accession numbers MT603999-MT604041 (Table 1).

Phylogenetic and genetic analyses were carried out by comparing nucleotide sequences of Thai CPIV-5 with those of PIV-5 available from the GenBank database. The reference nucleotide sequences of PIV-5 were retrieved based on geographic location, and host species including human PIV-1 (KF530221), swine PIV-1 (S033N; JX857410), human PIV-2 (NC003443), human PIV-3 (NC001796), swine PIV-3 (Texas-81; EU439429), and human PIV-4 (KF483663). Reference PIV-5 includes human strains (AGS; KX060176, DEN; JQ743322, MIL; JQ743326, MEL; JQ743325, RQ; JQ743327, LN; JQ743324), a rhesus macaque kidney cell strain (W3A; JQ743318.1), canine strains (HeN0718; KY114804, CC-14; KP893891, H221; JQ743323, 78524; JQ743319, CPI + ; JQ743321, CPI-; JQ743320, 08-1990; KC237063, D277; KC237065, 1168-1; KC237064), swine strains (SER; JQ743328, KNU-11; KC852177), a cattle strain (PV5-BC14; KM067467), a lesser panda strain (ZJQ-221; KX100034) and a pangolin strain (CAN; MH362816). Phylogenetic analysis of CPIV-5 was performed by using MEGA v.7.0 (Tempe, AZ, USA) with the neighbor-joining method with the Kimura 2-parameter with 1,000 bootstrap replicates^52^. For genetic analysis, the nucleotide sequences and deduced amino acids of CPIV-5 were aligned and compared using MegAlign software v.5.03 (DNASTAR Inc., Wisconsin, USA).

### Statistical analysis

Categorical data corresponding to the time of sample collection, age of dogs, and vaccination history were analyzed using the Fisher's exact test (https://www.socscistatistics.com/tests/fisher). A p-value of < 0.05 was considered as statistically significant.

### Ethics statement

This study was conducted under the approval of the Institute for Animal Care and Use Protocol of the CU-VET, Chulalongkorn University (IACUC # 1731074).

## Supplementary Information

Supplementary Information

## References

[1] Thomas SM, Lamb RA, Paterson RG (1988). Two mRNAs that differ by two nontemplated nucleotides encode the amino coterminal proteins P and V of the paramyxovirus SV5. Cell.

[2] Chew, F. T., Doraisingham, S., Ling, A. E., Kumarasinghe, G. & Lee, B. W. Seasonal trends of viral respiratory tract infections in the tropics. *Epidemiol. Infect.* **121**. 10.1017/s0950268898008905 (1998). 10.1017/s0950268898008905 PMC2809482 9747763

[3] Morgan OW (2013). Hospitalization due to human parainfluenza virus-associated lower respiratory tract illness in rural Thailand. Influenza Other Respir. Viruses.

[4] Ruampunpong H (2014). Human parainfluenza virus infection in Thai children with lower respiratory tract infection from 2010 to 2013. Southeast Asian J. Trop. Med. Public Health.

[5] Henrickson KJ (2003). Parainfluenza viruses. Clin. Microbiol. Rev..

[6] Hull, R. N., Minner, J. R. & Smith, J. W. New viral agents recovered from tissue cultures of monkey kidney cells. I. Origin and properties of cytopathogenic agents S.V.1, S.V.2, S.V.4, S.V.5, S.V.6, S.V.11, S.V.12 and S.V.15. *Am. J. Hyg.* **63**, 204–215 (1956). 10.1093/oxfordjournals.aje.a119804 13302209

[7] Chatziandreou N (2004). Relationships and host range of human, canine, simian and porcine isolates of simian virus 5 (parainfluenza virus 5). J. Gen. Virol..

[8] Basle M (1985). Paramyxovirus antigens in osteoclasts from Paget's bone tissue detected by monoclonal antibodies. J. Gen. Virol..

[9] Goswami K, Lange L, Mitchell D, Cameron K, Russell W (1984). Does simian virus 5 infect humans?. J. Gen. Virol..

[10] Goswami K, Randall R, Lange L, Russell W (1987). Antibodies against the paramyxovirus SV5 in the cerebrospinal fluids of some multiple sclerosis patients. Nature.

[11] Zhang L, Collins PL, Lamb RA, Pickles RJ (2011). Comparison of differing cytopathic effects in human airway epithelium of parainfluenza virus 5 (W3A), parainfluenza virus type 3, and respiratory syncytial virus. Virology.

[12] Danjoh I (2009). Is parainfluenza virus a threatening virus for human cancer cell lines?. Hum. Cell.

[13] Zhai JQ (2017). First complete genome sequence of parainfluenza virus 5 isolated from lesser panda. Arch. Virol..

[14] Lee YN, Lee C (2013). Complete genome sequence of a novel porcine parainfluenza virus 5 isolate in Korea. Arch. Virol..

[15] Liu Y (2015). Parainfluenza virus 5 as possible cause of severe respiratory disease in calves China. Emerg. Infect. Dis..

[16] Binn LN, Eddy GA, Lazar EC, Helms J, Murnane T (1967). Viruses recovered from laboratory dogs with respiratory disease. Proc. Soc. Exp. Biol. Med..

[17] Ellis JA, Krakowka GS (2012). A review of canine parainfluenza virus infection in dogs. J. Am. Vet. Med. Assoc..

[18] Joffe DJ (2016). Factors associated with development of Canine Infectious Respiratory Disease Complex (CIRDC) in dogs in 5 Canadian small animal clinics. Can. Vet. J..

[19] Viitanen SJ, Lappalainen A, Rajamaki MM (2015). Co-infections with respiratory viruses in dogs with bacterial pneumonia. J. Vet. Intern. Med..

[20] Ajiki M (1982). Isolation and characterization of parainfluenza 5 virus from a dog. Nihon Juigaku Zasshi.

[21] Baumgartner WK, Metzler AE, Krakowka S, Koestner A (1981). In vitro identification and characterization of a virus isolated from a dog with neurological dysfunction. Infect. Immun..

[22] Baumgartner WK, Krakowka S, Koestner A, Evermann J (1982). Acute encephalitis and hydrocephalus in dogs caused by canine parainfluenza virus. Vet. Pathol..

[23] Durchfeld B, Baumgartner W, Krakowka S (1991). Intranasal infection of ferrets (Mustela putorius furo) with canine parainfluenza virus. Zentralbl Veterinarmed B.

[24] Davidson WR, Appel MJ, Doster GL, Baker OE, Brown JF (1992). Diseases and parasites of red foxes, gray foxes, and coyotes from commercial sources selling to fox-chasing enclosures. J. Wildl. Dis..

[25] Chen Z (2012). Evaluating a parainfluenza virus 5-based vaccine in a host with pre-existing immunity against parainfluenza virus 5. PLoS ONE.

[26] Lazar EC, Swango LJ, Binn LN (1970). Serologic and infectivity studies of canine SV-5 virus. Proc. Soc. Exp. Biol. Med..

[27] Randall RE, Young DF, Goswami KK, Russell WC (1987). Isolation and characterization of monoclonal antibodies to simian virus 5 and their use in revealing antigenic differences between human, canine and simian isolates. J. Gen. Virol..

[28] Parisien JP, Lau JF, Horvath CM (2002). STAT2 acts as a host range determinant for species-specific paramyxovirus interferon antagonism and simian virus 5 replication. J. Virol..

[29] Ito M (2009). Effects of multiple amino acids of the parainfluenza virus 5 fusion protein on its haemagglutinin-neuraminidase-independent fusion activity. J. Gen. Virol..

[30] Bose S (2013). Mutations in the parainfluenza virus 5 fusion protein reveal domains important for fusion triggering and metastability. J. Virol..

[31] Rima BK (2014). Stability of the parainfluenza virus 5 genome revealed by deep sequencing of strains isolated from different hosts and following passage in cell culture. J. Virol..

[32] Monteiro FL (2016). Detection of respiratory viruses in shelter dogs maintained under varying environmental conditions. Braz. J. Microbiol..

[33] Mochizuki M, Yachi A, Ohshima T, Ohuchi A, Ishida T (2008). Etiologic study of upper respiratory infections of household dogs. J. Vet. Med. Sci..

[34] Ellis J (2011). Seroepidemiology of respiratory (group 2) canine coronavirus, canine parainfluenza virus, and Bordetella bronchiseptica infections in urban dogs in a humane shelter and in rural dogs in small communities. Can. Vet. J..

[35] Seyfiabad Shapouri, M. R., Avizeh, R., Mosallanejad, B. & Ramesh, B. Antigenic detection of Canine Parainfluenza virus in urban dogs with respiratory disease in Ahvaz area, southwestern Iran. *Arch. Razi Inst.* **64**, 115–120 (2016).

[36] Emery JB, House JA, Bittle JL, Spotts AM (1976). A canine parainfluenza viral vaccine: immunogenicity and safety. Am. J. Vet. Res..

[37] Erles K, Dubovi EJ, Brooks HW, Brownlie J (2004). Longitudinal study of viruses associated with canine infectious respiratory disease. J. Clin. Microbiol..

[38] Leser GP, Ector KJ, Lamb RA (1996). The paramyxovirus simian virus 5 hemagglutinin-neuraminidase glycoprotein, but not the fusion glycoprotein, is internalized via coated pits and enters the endocytic pathway. Mol. Biol. Cell.

[39] Robach JG, Lamb RA (2010). Analysis of parainfluenza virus-5 hemagglutinin-neuraminidase protein mutants that are blocked in internalization and degradation. Virology.

[40] Melanson VR, Iorio RM (2004). Amino acid substitutions in the F-specific domain in the stalk of the newcastle disease virus HN protein modulate fusion and interfere with its interaction with the F protein. J. Virol..

[41] Yuan P (2005). Structural studies of the parainfluenza virus 5 hemagglutinin-neuraminidase tetramer in complex with its receptor, sialyllactose. Structure.

[42] Baty DU, Randall RE (1993). Multiple amino acid substitutions in the HN protein of the paramyxovirus, SV5, are selected for in monoclonal antibody resistant mutants. Arch. Virol..

[43] Corey EA, Iorio RM (2007). Mutations in the stalk of the measles virus hemagglutinin protein decrease fusion but do not interfere with virus-specific interaction with the homologous fusion protein. J. Virol..

[44] Timani KA (2008). A single amino acid residue change in the P protein of parainfluenza virus 5 elevates viral gene expression. J. Virol..

[45] Sun D, Luthra P, Xu P, Yoon H, He B (2011). Identification of a phosphorylation site within the P protein important for mRNA transcription and growth of Parainfluenza Virus 5. J. Virol..

[46] Sun D, Xu P, He B (2011). Sumoylation of the P protein at K254 plays an important role in growth of parainfluenza virus 5. J. Virol..

[47] Wilson RL (2006). Function of small hydrophobic proteins of paramyxovirus. J. Virol..

[48] He B, Lin GY, Durbin JE, Durbin RK, Lamb RA (2001). The SH integral membrane protein of the paramyxovirus simian virus 5 is required to block apoptosis in MDBK cells. J. Virol..

[49] Posuwan N (2010). Prevalence of respiratory viruses isolated from dogs in Thailand during 2008–2009. Asian Biomed..

[50] Liu C (2017). Isolation and genomic characterization of a canine parainfluenza virus type 5 strain in China. Arch. Virol..

[51] Rozen S, Skaletsky H (2000). Primer3 on the WWW for general users and for biologist programmers. Methods Mol. Biol..

[52] Tamura K, Stecher G, Peterson D, Filipski A, Kumar S (2013). MEGA6: molecular evolutionary genetics analysis version 6.0. Mol. Biol. Evol..

