# Supplementary material for: Molecular detection and whole genome characterization of Canine Parainfluenza type 5 in Thailand
Source: Sci Rep. 2021 Feb 16;11:3866. doi: 10.1038/s41598-021-83323-9 (PMC7887266; doi:10.1038/s41598-021-83323-9)
Supplement: Supplementary file 1 — Supplementary Information [file 41598_2021_83323_MOESM1_ESM.docx]

**Supplementary Material**

**Molecular detection and whole genome characterization of**

**Canine Parainfluenza type 5 in Thailand**

Kamonpan Charoenkul^1,2^, Chanakarn Nasamran^1,2^, Taveesak Janetanakit^1,2^, Supassama Chaiyawong^1,2^, Napawan Bunpapong^1,3^, Supanat Boonyapisitsopa^1^, Ratanaporn Tangwangvivat^1^ and Alongkorn Amonsin^1,2*^

^1^ Center of Excellence for Emerging and Re-emerging Infectious Diseases in Animals, Faculty of Veterinary Science, Chulalongkorn University, Bangkok, Thailand

^2^ Department of Veterinary Public Health, Faculty of Veterinary Science, Chulalongkorn University, Bangkok, Thailand

^3^ Veterinary Diagnostic Laboratory, Faculty of Veterinary Science, Chulalongkorn University, Bangkok, Thailand

*Corresponding author: Professor Dr. Alongkorn Amonsin

Mailing address: Department of Veterinary Public Health, Faculty of Veterinary Science, Chulalongkorn University, Bangkok, Thailand 10330

Phone: +66 2218 9578 Fax: +66 2218 9577

E-mail: Alongkorn.a@chula.ac.th

**Keywords:** Characterization; Dogs; Parainfluenza type 5; Thailand

Running Head:

Characterization of CPIV-5 in Thailand

**Supplement tables**

Supplement Table 1. Details of the sample collection and detection of CPIV-5

Supplement Table 2. Genetic analysis of the F gene of Thai CPIV-5 and reference PIV

Supplement Table 3. Genetic analysis of the V/P, SH and non-coding region of Thai CPIV-5 and reference PIV-5

Supplement Table 4. Nucleotide sequences of primers used for CPIV-5 detection and

sequencing in this study

Supplement Table 1. Details of the sample collection and detection of CPIV- 5

| Year | Month | Total samples | Number positive for CPIV5 (%) |
| --- | --- | --- | --- |
| 2015 | Nov | 7 | 0 |
|  | Dec | 10 | 0 |
| 2016 | Jan | 9 | 1 (11.1%) |
|  | Feb | 11 | 1 (9.1%) |
|  | Mar | 7 | 0 |
|  | Apr | 13 | 1 (7.7%) |
|  | May | 12 | 1 (8.3%) |
|  | June | 3 | 0 |
|  | July | 15 | 0 |
|  | Aug | 10 | 0 |
|  | Sep | 9 | 0 |
|  | Oct | 11 | 1 (9.1%) |
|  | Nov | 12 | 5 (41.7%)* |
|  | Dec | 15 | 5 (33.3%)** |
| 2017 | Jan | 12 | 3 (25.0%) |
|  | Feb | 26 | 0 |
|  | Mar | 32 | 5 (15.6%) |
|  | Apr | 24 | 0 |
|  | May | 11 | 0 |
|  | June | 11 | 0 |
|  | July | 19 | 0 |
|  | Aug | 17 | 0 |
|  | Sep | 23 | 1 (4.4%) |
|  | Oct | 21 | 0 |
|  | Nov | 13 | 0* |
|  | Dec | 33 | 3 (9.1%) |
| 2018 | Jan | 19 | 1 (5.3%) |
|  | Feb | 17 | 2 (11.8%) |
|  | Mar | 14 | 0 |
|  | Apr | 18 | 0 |
|  | May | 9 | 1 (11.1%) |
|  | June | 19 | 0 |
|  | July | 21 | 0 |
|  | Aug | 18 | 0 |
|  | Sep | 11 | 1 (9.1%) |
|  | Oct | 10 | 0 |
|  | Nov | 15 | 0* |
|  | Dec | 14 | 0** |
|  | Total | 571 | 1. 5.60%)38 |

*Fisher exact test; p=0.0098-0.0149 (p <0.05) statistical significance when compared to the same month of other years

**Fisher exact test; p=0.042 (p <0.05) statistical significance when compared to the same month of other years

Supplement Table 2. Genetic analysis of the F gene of Thai CPIV-5 and reference PIV

| Virus | **Host** |  |  | **Primate specific amino acid** | | | | | | |
| --- | --- | --- | --- | --- | --- | --- | --- | --- | --- | --- |
|  |  | **L22P** | **S443P** | **3** | **19** | **310** | **438** | **498** | **530** | **536** |
| **Reference PIV-5** |  |  |  |  |  |  |  |  |  |  |
| DEN | Human | P | P | I | G | M | S | F | Q | Q |
| MIL | Human | P | P | I | G | M | S | F | Q | Q |
| MEL | Human | P | P | I | G | M | S | F | - | - |
| RQ | Human | P | P | I | G | M | S | F | Q | Q |
| LN | Human | P | P | I | G | M | S | F | Q | Q |
| AGS | Human | P | P | T | S | M | S | L | Q | Q |
| W3A | Macaque cell | P | S | T | S | M | S | L | - | - |
| SER | Swine | P | P | T | S | I | T | L | S | R |
| KNU-11 | Swine | L | P | T | S | I | T | L | S | R |
| HLJ2015_DP1-1 | Swine | P | P | T | S | I | T | L | S | R |
| PV5-BC14 | Calve | L | P | T | S | I | T | L | S | R |
| ZJQ-221 | Lesser panda | P | P | T | S | I | T | L | S | R |
| CC-14 | Canine | L | P | T | S | I | T | L | S | R |
| H221 | Canine | P | P | T | S | I | T | L | S | R |
| 78524 | Canine | P | P | T | S | I | T | L | S | R |
| CPI+ | Canine | P | P | T | S | I | T | L | S | R |
| CPI- | Canine | P | P | T | S | I | T | L | S | R |
| 08-1990 | Canine | P | P | T | S | I | T | L | S | R |
| D277 | Canine | P | P | T | S | I | T | L | S | R |
| 1168-1 | Canine | P | P | T | S | I | T | L | S | R |
| HeN0718 | Canine | P | P | T | S | I | T | L | S | R |
|  |  |  |  |  |  |  |  |  |  |  |
| **This study** |  |  |  |  |  |  |  |  |  |  |
| CU-D58 | Canine | P | P | T | S | I | T | L | S | R |
| CU-D103 | Canine | P | P | T | S | I | T | L | S | R |
| CU-D133 | Canine | P | P | T | S | I | T | L | S | R |
| CU-D151 | Canine | P | P | T | S | I | T | L | S | Q |
| CU-D373 | Canine | P | P | T | S | I | T | L | S | R |
| CU-D376 | Canine | P | P | T | S | I | T | L | S | R |
| CU-D381 | Canine | P | P | T | S | I | T | L | S | R |
| CU-D399 | Canine | P | P | T | S | I | T | L | S | R |
| CU-D400 | Canine | P | P | T | S | I | T | L | S | R |
| CU-D406 | Canine | P | P | T | S | I | T | L | S | R |
| CU-D466 | Canine | P | P | T | S | I | T | L | S | R |
| CU-D585 | Canine | P | P | T | S | I | T | L | S | R |
| CU-D20804 | Canine | P | P | T | S | I | T | L | S | R |

Supplement Table 3. Genetic analysis of the V/P, SH and non-coding region of Thai CPIV-5 and reference PIV-5

| Virus | Host |  | V/P |  |  |  | SH | Insertion at position 6506-6511 (6-nt) | Insertion at position 8394  (1-nt) |
| --- | --- | --- | --- | --- | --- | --- | --- | --- | --- |
|  |  | S157F | T286A | K254R | T293K | S308A | Start codon |  |  |
| **Reference PIV-5** | | | | | | | | | |
| AGS | AGS Cell | S | T | K | T | S | M | No | A |
| DEN | Human | F | T | K | T | S | M | No | No |
| MIL | Human | F | T | K | T | S | M | No | No |
| MEL | Human | F | T | K | T | S | M | No | No |
| RQ | Human | F | T | K | T | S | M | AAAGAA | No |
| LN | Human | F | T | K | T | S | M | AAAGAA | No |
| W3A | Macaque cell | S | T | K | T | S | M | No | No |
| SER | Swine | S | T | K | K | S | M | No | No |
| KNU-11 | Swine | S | T | K | K | S | T | No | No |
| PV5-BC14 | Calve | S | T | K | K | S | T | No | No |
| ZJQ-221 | Lesser panda | S | T | K | K | S | T | No | No |
| CC-14 | Canine | S | T | K | K | S | T | No | No |
| H221 | Canine | S | T | K | K | S | M | No | No |
| 78524 | Canine | F | T | K | K | S | M | No | No |
| CPI+ | Canine | F | T | K | K | S | T | No | No |
| CPI- | Canine | F | T | K | K | S | T | No | No |
| 08-1990 | Canine | S | T | K | K | S | M | No | No |
| D277 | Canine | S | T | K | K | S | M | No | No |
| 1168-1 | Canine | S | T | K | K | S | M | No | No |
|  |  |  |  |  |  |  |  |  |  |
| **This study** | | | | | | | | | |
| CU-D58 | Canine | S | T | K | K | S | T | N/A | N/A |
| CU-D103 | Canine | S | T | K | K | S | T | N/A | N/A |
| CU-D133 | Canine | S | T | K | K | S | T | No | No |
| CU-D151 | Canine | S | T | K | K | S | T | No | No |
| CU-D373 | Canine | S | T | K | K | S | M | N/A | N/A |
| CU-D376 | Canine | S | T | K | K | S | T | N/A | N/A |
| CU-D381 | Canine | S | T | K | K | S | T | N/A | N/A |
| CU-D399 | Canine | S | T | K | K | S | M | N/A | N/A |
| CU-D400 | Canine | S | T | K | K | S | M | N/A | N/A |
| CU-D406 | Canine | S | T | K | K | S | T | N/A | N/A |
| CU-D466 | Canine | S | T | K | K | S | M | N/A | N/A |
| CU-D585 | Canine | S | T | K | K | S | M | N/A | N/A |
| CUD20804 | Canine | S | T | K | K | S | T | No | No |

Supplement Table 4. Nucleotide sequences of primers used for CPIV-5 detection and sequencing in this study

| **Primer name** | **Forward (5’-3’)** | **Primer name** | **Reward (5’-3’)** | **Position *** | **Gene** | **Product size** | **Reference** |
| --- | --- | --- | --- | --- | --- | --- | --- |
| **Primer detection** | |  |  |  |  |  |  |
| CPiV- F363 | GGGTAGAGATCGATGGCTTTGA | CPiV-R538 | GCGCAGTCATGCACTTGCAAGT |  | NP | 188 | Posuwan, N. *et al*, 2010 |
| CPiV-F428 | GCCGTGGAGAGATCAATGCCTAT |  |  |  |  |  |  |
| **Primer sequencing** | |  |  |  |  |  |  |
| PIV5_ 1F | AGGGGAAAATGAAGTGGTGA | PIV5_ 1R | AGCCTATTCCCATAGCATAGCT | 5-1173 | NP | 1168 | This study |
| PIV5_ 2F | GGCTCGACGAATAATCCAGA | PIV5_ 2R | GCGGCATTCAAGTCATCTGT | 813-1679 | NP | 866 | This study |
| PIV5_ 3F | GGTCTAACTCAAGCCGAACG | PIV5_ 3R | TCGGGGTGGAGTCCAGACC | 1324-2194 | NP | 870 | This study |
| PIV5_ 4F | CGGACGGGTTAGTAACAAGC | PIV5_ 4R | TCCCTGCTTTGAGTCCAACT | 1814-2657 | V/P | 843 | This study |
| PIV5_ 5F | CAGTTCCCCAACCGATTTTA | PIV5_ 5R | TGACAAAAATGTCGGAATGAA | 2334-3683 | V/P | 1349 | This study |
| PIV5_ 6F | CGACCAACTGCAACAAGTG | PIV5_ 6R | TCTCCACGAGAAGAGAGATGC | 2835-4176 | M | 1341 | This study |
| PIV5_ 7F | CCACTGGTGACGTTCGTAAA | PIV5_ 7R | AAGTTGCCGGACATTTGTTG | 3311-4663 | M | 1352 | This study |
| PIV5_ 8F | AGGTCCACATCCCTGACAAG | PIV5_ 8R | TGCGGGGTTTGTAATTTGAT | 3816-5194 | F | 1378 | This study |
| PIV5_ 9F | TCGAGATTACACCACCTCAAA | PIV5_ 9R | TCCATCAAACAGCACGAATC | 4307-5671 | F | 1364 | This study |
| PIV5_ 10F | GTTGATTCCAACTCGGAGGA | PIV5_ 10R | TGAGATCAGACCGTGAGTGG | 4828-6167 | F | 1339 | This study |
| PIV5_ 11F | GGCCAGATTGTGGGATTAGA | PIV5_ 11R | TCGAAATAATACTCGGCAAGTG | 5318-6656 | F, SH | 1338 | This study |
| PIV5_ 12F | AACCTACAATAGCACCATCAAGC | PIV5_ 12R | ACATGATCCTGGCATCCATT | 5836-7169 | HN | 1333 | This study |
| PIV5_ 13F | CATTGTCGCTGCTAATCGAA | PIV5_ 13R | TTCTGGAGGAGCGGTAGAAA | 6338-7679 | HN | 1341 | This study |
| PIV5_ 14F | CTCTGCAGTCGCTCTACCTCT | PIV5_ 14R | CTTGACCGCTTGATCCAAAT | 6838-8161 | HN | 1323 | This study |
| PIV5_ 15F | TCAACCAGAGAGGGATGACTAC | PIV5_ 15R | GCCAATGGCCCTTTCTAAG | 7338-8679 | HN | 1341 | This study |
| PIV5_ 16F | TGGCCTATGACCATGCTGTA | PIV5_ 16R | AAAATGTTGTGACGACCTTCG | 7831-9189 | L | 1358 | This study |
| PIV5_ 17F | CAGGTCTGACCCAGCAAATC | PIV5_ 17R | GGCCATACTCCATGATGCTT | 8315-9689 | L | 1374 | This study |
| PIV5_ 18F | AATGCGGGTACTTCCCAGTG | PIV5_ 18R | CCAGCATGGTTCGCTAAAAG | 8809-10187 | L | 1378 | This study |
| PIV5_ 19F | ATGTCAGATCCCATCCCAGA | PIV5_ 19R | AGCCCTTCAATTCCACCTCT | 9340-10658 | L | 1318 | This study |
| PIV5_ 20F | CGCAGGTGAGGAACTCAGTA | PIV5_ 20R | TCCAGGAATTGACACTTGAGG | 9828-11173 | L | 1345 | This study |
| PIV5_ 21F | AATCACGTCACTCCAAGCAA | PIV5_ 21R | TCTGTTTTCTCCTCCCGACA | 10343-11656 | L | 1313 | This study |
| PIV5_ 22F | TTGAGAAAAAGACTATTGCTTTTAGA | PIV5_ 22R | TACACTCCTGCCAGTCTAAGA | 10808-12178 | L | 1370 | This study |
| PIV5_ 23F | TTCCGCAGTTGCAGATCTTA | PIV5_ 23R | GCGGGTCTTCATCAAATACAA | 11307-12656 | L | 1349 | This study |
| PIV5_ 24F | AGCATCGACATTGCAAGGAG | PIV5_ 24R | TCCATTGGAGAGCACATTGA | 11842-13194 | L | 1352 | This study |
| PIV5_ 25F | GGATGATGGCACAACTACCC | PIV5_ 25R | CTCCAAGTTTGCATTGGACTC | 12315-13677 | L | 1362 | This study |
| PIV5_ 26F | TGCCATTGTTGCATCAGACT | PIV5_ 26R | AAGCTTGCACTTGACTCCAAA | 12825-14187 | L | 1362 | This study |
| PIV5_ 27F | AAGGGGTTCTCTCCTGATGA | PIV5_ 27R | AAGTTGCGACTGGCTCGATA | 13324-14674 | L | 1350 | This study |
| PIV5_ 28F | GCTACCACAGGGTGATCATTT | PIV5_ 28R | TATTTAGATTTCCTCGCCATCG | 13828-15206 | L | 1378 | This study |

* Position based on CPIV-5 strain 08-1990 (KC237063)
